# Understanding factors impacting patient-reported outcome measures integration in routine clinical practice: an umbrella review

**DOI:** 10.1007/s11136-024-03728-7

**Published:** 2024-07-18

**Authors:** Michael Anderson, Robin van Kessel, Eleanor Wood, Adam Stokes, Jon Fistein, Ian Porter, Elias Mossialos, Jose M. Valderas

**Affiliations:** 1 Health Organisation, Policy, Economics (HOPE), Centre for Primary Care & Health Services Research, The University of Manchester, Manchester, UK; 2 LSE Health, Department of Health Policy, London School of Economics and Political Science, London, UK; 3 Department of International Health, Care and Public Health Research Institute (CAPHRI), Faculty of Health, Medicine and Life Sciences, Maastricht University, Maastricht, The Netherlands; 4 Centre for Global Health, St Georges, University of London, London, UK; 5 Department of Public Health and Primary Care, University of Cambridge, Cambridge, UK; 6 Health Services and Policy Research Group, University of Exeter, Exeter, UK; 7 Institute of Global Health Innovation, Imperial College London, London, UK; 8 Centre for Research On Health Systems Performance, National University of Singapore, Singapore, Singapore

**Keywords:** Patient reported outcome measures, PROMs, Outcome measurement, Implementation, Theoretical domains framework

## Abstract

**Purpose:**

Patient-report outcome measures (PROMs) have gained widespread support as a mechanism to improve healthcare quality. We aimed to map out key enablers and barriers influencing PROMs implementation strategies in routine clinical practice.

**Methods:**

An umbrella review was conducted to identify reviews exploring enablers and barriers related to the integration of PROMs in routine clinical practice from January 2000 to June 2023. Information on key enablers and barriers was extracted and summarised thematically according to the Theoretical Domains Framework.

**Results:**

34 reviews met our criteria for inclusion. Identified reviews highlighted barriers such as limited PROMs awareness among clinicians and patients, perceived low value by clinicians and patients, PROMs that were too complex or difficult for patients to complete, poor usability of PROMs systems, delayed feedback of PROMs data, clinician concerns related to use of PROMs as a performance management tool, patient concerns regarding privacy and security, and resource constraints. Enablers encompassed phased implementation, professional training, stakeholder engagement prior to implementation, clear strategies and goals, ‘change champions’ to support PROMs implementation, systems to respond to issues raised by PROMs, and integration into patient pathways. No consensus favoured paper or electronic PROMs, yet offering both options to mitigate digital literacy bias and integrating PROMs into electronic health records emerged as important facilitators.

**Conclusions:**

The sustainable implementation of PROMs is a complex process that requires multicomponent organisational strategies covering training and guidance, necessary time and resources, roles and responsibilities, and consultation with patients and clinicians.

**Supplementary Information:**

The online version contains supplementary material available at 10.1007/s11136-024-03728-7.

## Introduction

Patient-reported outcome measures (PROMs) are valuable tools for assessing a patient’s health status and well-being, providing valuable information on patients’ quality of life, symptoms, and functioning [1–3]. Originally conceived as a research tool to facilitate the measurement of more subjective health outcomes, their potential value in clinical practice and as a mechanism to improve healthcare quality and promote patient-centred healthcare delivery has been increasingly recognised [4–7]. However, despite their potential benefits, the implementation of PROMs has been challenging, with adoption rates remaining low [8]. Many procedure and speciality-specific PROMs have been developed and implemented, yet response rates and completion rates vary substantially. For example, an international review of published registry-based studies on PROMs with at least two follow-up time points found response rates varied from 100% to less than 30% [9].

Several key previous reviews have focused on different aspects related to the use of PROM in routine clinical practice. Greenhalgh et al. summarised the findings of two related realist syntheses focused on the feedback of aggregate and individual-level PROMs data to improve patient care [10]. They found that providers were more likely to take steps to improve patient care if PROMs data were perceived as credible, gave a clear indication of the source of problems, and feedback took place in a timely manner. They emphasised the need for more support and guidance for providers regarding collection and interpretation of PROMs data, and emphasised how tensions between the use of PROMs as a quality improvement tool and to support individual patients may negatively influence implementation. While the realist review approach used by Greenhalgh et al. is an effective way to identify ideas and assumptions regarding how PROMs are used in routine clinical practice, this approach does not comprehensively address how to overcome issues that prevent the consistent implementation of PROMs in routine clinical practice.

Gibbons et al. examined the effect of PROMs feedback to patients, or healthcare workers, on quality of care. [11] They identified 116 randomised trials which evaluated the effects of using PROMs in routine clinical practice in a variety of clinical settings including primary care, psychiatry, and oncology contexts. Overall, they found evidence that the use of PROMs in routine clinical practice improves quality of life, and increases patient-clinician communication, diagnosis of disease, and disease control. While this review provides promising evidence regarding the value of using PROMs in routine clinical practice, the review does not provide an assessment of how to encourage consistent implementation of PROMs in routine clinical practice to maximise this value.

Foster et al. focused on implementation of PROMs in routine clinical practice and mapped enablers and barriers to implementation identified in 6 reviews using the Consolidated Framework for Implementation Research (CFIR). They highlighted challenges in PROMs utilization within routine clinical practice, such as resource constraints, questionnaire complexity, data interpretation hurdles, and professional reluctance [12]. Using an implementation science framework approach has advantages as it incorporates a structured approach to implementation of complex healthcare interventions that can be used to map multiple stakeholder perspectives across different implementation stages [13]. However, multiple implementation science frameworks exist and have been shown to produce different results when examining the same issue. [14, 15] Therefore, there is a need to apply alternative implementation science frameworks beyond the CFIR to understand PROMs implementation in routine clinical practice to ascertain if this generates further insights. Moreover, the scientific literature around PROMs implementation in clinical practice has rapidly expanded in recent years and there is a need to undertake an updated overview of reviews to consolidate insights from more recent reviews. This is also important as there has been rapid digitalisation of healthcare services over the last 5 years, significantly accelerated by the emergence of the COVID-19 pandemic [16]. This has created further opportunities to integrate PROMs within electronic healthcare records, [17–20] which may influence the enablers and barriers to PROMs implementation in routine clinical practice. To address these unmet needs, we set forth to conduct a synthesis of the literature to map out key enablers and barriers influencing PROMs implementation in routine clinical practice.

## Methods

We conducted an umbrella review in accordance to methods contained within a previously published protocol [21]. An umbrella review is defined as a systematic collection and assessment of multiple systematic reviews and meta-analyses on a specific research topic [22]. Joanna Briggs Institute (JBI) guidelines for umbrella reviews were followed in the design and execution of the research, [23] and the PRISMA guidelines for reporting purposes [24]. An umbrella review was utilised as it was perceived as a feasible way to undertake a review with a broad focus encompassing a large body of literature.

### Eligibility criteria

The inclusion criteria for this review were: (1) any type of literature review using a systematic search (e.g., systematic reviews, realist reviews, and scoping reviews); (2) a focus on enablers and barriers to the implementation of PROMs in routine clinical practice; (3) published in English; and (4) published between January 2000 and June 2023. While we imposed no exclusion criteria based on PROM type or clinical speciality, we did exclude narrative reviews, as well as reviews focusing on PROMs application in clinical trials and those centred on the validation or measurement properties of PROMs. These exclusions were made due to the primary focus of our review on the process of implementing PROMs into routine clinical practice. We defined “routine clinical practice” as a health service setting providing patient care such as primary health clinics, hospital outpatient clinics or specialist medical centres. [25]

### Search strategy and data collection

We searched for relevant reviews published between January 2000 and December 2020 using Ovid to search the MEDLINE, EMBASE, and PsychINFO databases. We chose to limit our search strategy to three databases for feasibility purposes and because research has indicated that searching at least two databases improves coverage and recall, and decreases risk of missing eligible studies [26]. We restricted our search period to include reviews published from the year 2000 onwards to ensure findings from our umbrella review were relevant to current routine clinical practice, as reviews published before this date may reflect contexts and environments with outdated healthcare provision. Moreover, Foster et al. had a similar eligibility criteria and did not identify any reviews before 2000 [12]. Aiming for a broad yet sensitive search strategy, we developed a simple search strategy and the following search terms which was applied by Ovid to each database: ((“patient reported outcome” OR “patient reported outcomes” OR “PROM” OR “PROMS”) AND (“implement*” OR “barrier*” OR “facilitat*” OR “enabl*”)).ab, ti. Two primary reviewers (MA, AS) independently screened articles to identify relevant reviews, and then reviewed the full text of articles to assess if they met eligibility criteria. To ensure our umbrella review included more recent reviews prior to submission for publication, the search was later repeated and extended to June 2023 by two primary reviewers (MA, EW). A third reviewer (IP) resolved any disagreements between the two reviewers. We searched the reference list of all identified reviews and surveyed co-authors to suggest additional reviews not captured by our search strategy to identify other relevant reviews to include within our analysis. We also supplemented our search through grey literature by searching Google Scholar and reviewing the first 200 results as recommended by Haddaway et al. [27]

Quality assessment was conducted by two primary reviewers (MA, EW) using the Critical Appraisal Skills Programme (CASP) systematic review checklist, [28] and then discussed together to reach a collective judgement on the quality of each review. This tool was specifically designed for the assessment of a range of dimensions of quality in systematic reviews, including whether the review addresses a clearly focused question, included all relevant studies, and assessed potential bias of included studies.

### Data extraction and thematic analysis

Data extraction and thematic analysis was conducted through multiple phases. First, the two primary reviewers (MA and EW) independently extracted relevant enablers and barriers to PROMs implementation from each review that met our eligibility criteria. The following information was also summarised for each review: the focus of the review including relevant clinical speciality, type of review, approach to data synthesis categorised according to Barnett-Page and Thomas, [29] and perspectives captured. Second, these reviewers then met to discuss each implementation enabler and barrier identified to consolidate them into distinct factors and minimise duplication and overlap. Finally, these reviewers clustered the final set of implementation enablers and barriers using constructs contained within the Theoretical Domains Framework (TDF) for the thematic analysis (Table 1). We chose to use the TDF as it provides a behavioural science perspective targeted towards identification of enablers and barriers to the implementation of complex healthcare interventions from the perspective of relevant stakeholders involved [30–32], and has previously been applied to understand implementation challenges for PROMs specifically several times [14, 15, 33]. Application of a behavioural science approach is particularly useful to understand as it can help identify the motivations, beliefs, and incentives that drive behaviours that may encourage or hinder implementation of complex healthcare interventions [34]. The second and third phase was achieved through iterative discussions between the two reviewers until consensus was reached. Analysis was deductive in that the domains of the TDF were used as predetermined themes [35]. This approach to data synthesis was taken to ensure enablers and barriers to PROMs implementation were presented in a structured manner and has been validated by previous research [36].

**Table 1:** Theoretical Domains Framework

Domain	Construct
1. Knowledge (An awareness of the existence of something)	Knowledge (including knowledge of condition/scientific rationale)
	Procedural knowledge
	Knowledge of task environment
2. Skills (An ability or proficiency acquired through practice)	Skills
	Skills development
	Competence
	Ability
	Interpersonal skills
	Practice
	Skill assessment
3. Social influences/professional role and identity (A coherent set of behaviours and displayed personal qualities of an individual in a social or work setting)	Professional identity
	Professional role
	Social identity
	Identity
	Professional boundaries
	Professional confidence
	Group identity
	Leadership
	Organisational commitment
4. Beliefs about capabilities (Acceptance of the truth, reality or validity about an ability, talent or facility that a person can put to constructive use)	Self-confidence
	Perceived competence
	Self-efficacy
	Perceived behavioural control
	Beliefs
	Self-esteem
	Empowerment
	Professional confidence
5. Optimism (The confidence that things will happen for the best or that desired goals will be attained)	Optimism
	Pessimism
	Unrealistic optimism
	Identity
6. Beliefs about Consequences (Acceptance of the truth, reality, or validity about outcomes of a behaviour in a given situation)	Beliefs
	Outcome expectancies
	Characteristics of outcome expectancies
	Anticipated regret
	Consequents
7. Reinforcement (Increasing the probability of a response by arranging a dependent relationship, or contingency, between the response and a given stimulus)	Rewards (proximal/distal, valued/not valued, probable/improbable)
	Incentives
	Punishment
	Consequents
	Reinforcement
	Contingencies
	Sanctions
8. Intentions (A conscious decision to perform a behaviour or a resolve to act in a certain way)	Stability of intentions
	Stages of change model
	Transtheoretical model and stages of change
9. Goals (Mental representations of outcomes or end states that an individual wants to achieve)	Goals (distal/proximal)
	Goal priority
	Goal/target setting
	Goals (autonomous/controlled)
	Action planning
	Implementation intention
10. Memory, attention and decision processes (The ability to retain information, focus selectively on aspects of the environment and choose between two or more alternatives)	Memory
	Attention
	Attention control
	Decision making
	Cognitive overload/tiredness
11. Environmental context and resources (Any circumstance of a person’s situation or environment that discourages or encourages the development of skills and abilities, independence, social competence and adaptive behaviour)	Environmental stressors
	Resources/material resources
	Organisational culture/climate
	Salient events/critical incidents
	Person × environment interaction
	Barriers and facilitators
12. Social influences (Those interpersonal processes that can cause individuals to change their thoughts, feelings, or behaviours)	Social pressure
	Social norms
	Group conformity
	Social comparisons
	Group norms
	Social support
	Power
	Intergroup conflict
	Alienation
	Group identity
	Modelling
13. Emotion (A complex reaction pattern, involving experiential, behavioural, and physiological elements, by which the individual attempts to deal with a personally significant matter or event)	Fear
	Anxiety
	Affect
	Stress
	Depression
	Positive/negative affect
	Burn-out
14. Behavioural regulation (Anything aimed at managing or changing objectively observed or measured actions)	Self-monitoring
	Breaking habit
	Action planning

Source: Atkins et al. [35]

## Results

**Fig. 1:**
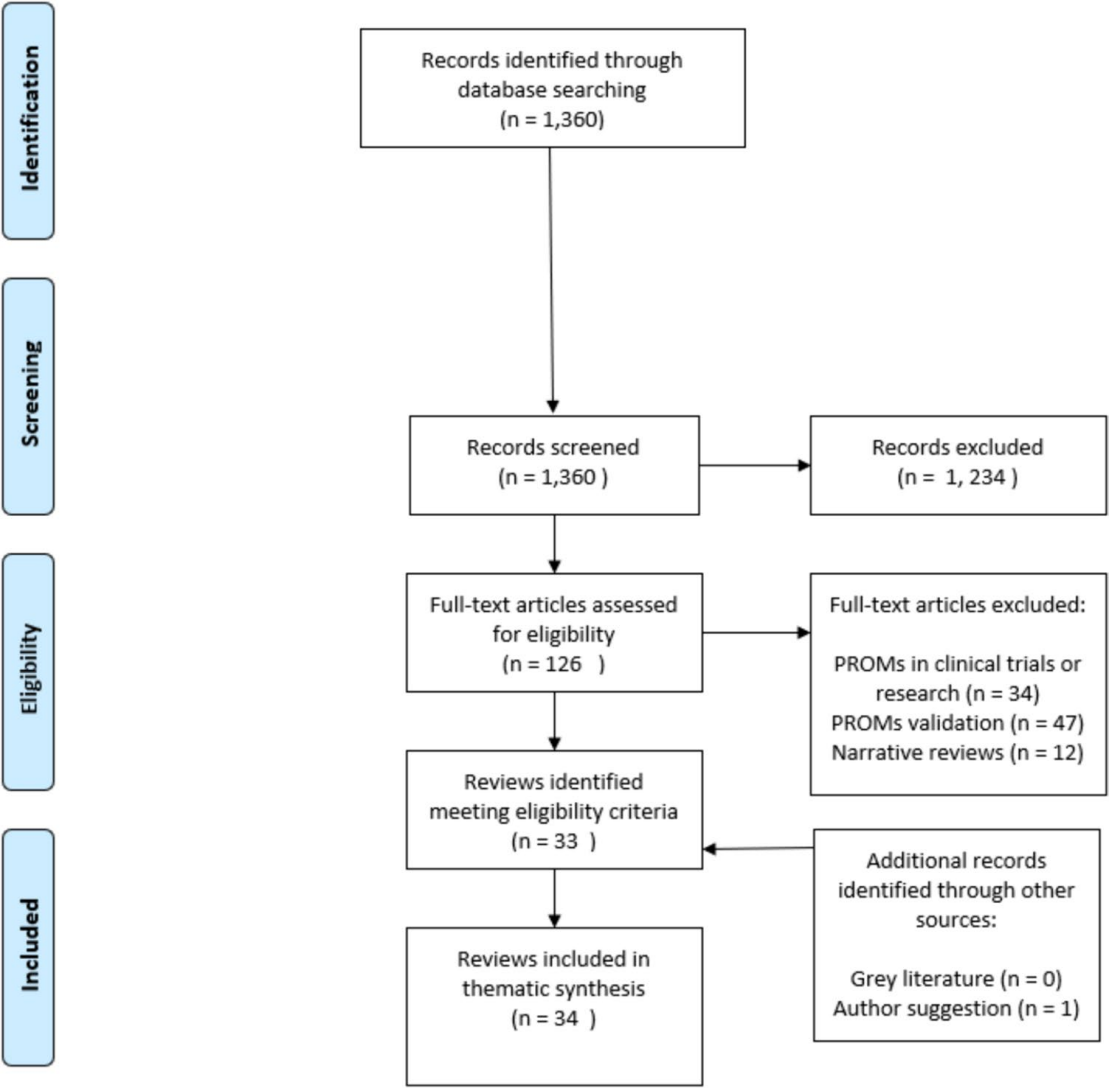
PRISMA flow diagram for identification, screening, eligibility and inclusion of reviews

In total, the search yielded 1360 results (Fig. 1). After screening, 126 reviews were identified for full text screening. After full-text screening, we identified 33 reviews that met our eligibility criteria. One additional review was identified after being suggested by a co-author of this review (MA).

Of the 34 identified reviews, 18 were systematic reviews (Supplementary Material File 1). Other approaches used included scoping reviews (8), realist reviews (4), umbrella reviews (2), an integrative review (1), and a systematic mapping study (1). The focus of each review varied significantly, with many reviews focusing on implementation challenges in specific settings including palliative care, [37] physical rehabilitation, [38] mental health services, [39] oncology, [40, 41] cancer care, [42–49] orthopaedics, [50] surgical practice [51] and paediatrics [52]. There were also reviews focusing on specific implementation challenges including alternative approaches to displaying PROMs data, [53] ePROM use [54], aggregated PROMs, [55] and the interpretation and use of PROMs data [10]. The quality of reviews varied with some reviews not addressing clearly focused research questions (*n* = 7), not including comprehensive methodologies to capture all relevant studies (*n* = 9), or not having quality assessments in place (*n* = 16). In many cases, negative quality scores regarding unclear research questions or methodological limitations were because reviews had broad research questions related to PROMs implementation that did not have clearly defined outcomes. No reviews were excluded from our narrative thematic analysis based on quality assessment as it was felt this would exclude significant and relevant information. A summary of identified reviews, approaches to data synthesis, perspectives captured, and quality issues is contained in Supplementary Material File 1, and the full results of our quality assessment is contained within Supplementary Material File 2.

Enablers and barriers to PROMs implementation in routine clinical practice were identified for all 14 construct within the TDF. We outline below how each domain can be understood in relation to PROMs implementation, and then discuss how findings from identified reviews relate to each domain. In some cases, certain enablers and barriers were relevant to more than one TDF. When possible we also describe the relevance of each enabler and barrier to behaviour change for relevant stakeholders involved in PROM implementation (i.e. patients, clinicians, and managers). In some cases, reviews describe system-level enablers and barriers to PROMs implementation in routine clinical practice without specifying their relevance to specific stakeholders. Findings are also summarised within Table 2.

**Table 2:** An overview of the enablers and barriers to implementing PROMs extracted from the included articles

Domain	Enablers	Barriers
1. Knowledge	Perceived high awareness about PROMs among clinicians and patients, and their potential impact on quality was persistently highlighted as an enabler or barrier to improving the implementation of PROMs [10, 12, 37–39, 42, 45, 56]. Consultation with all relevant stakeholders, including managers, clinicians, patients, to improve awareness and develop a strategy for local implementation [12, 39, 41, 44, 52, 54].	Low awareness about PROMs among clinicians and patients, and their potential impact on quality [10, 12, 37, 38, 42, 45, 49, 56].
2. Skills	Clinicians trialling PROMs before their introduction, to improve skills [12, 39, 44, 52]. Conducting a trial—potentially with patients—could assist implementation as the organisation could formulate a bespoke system [12, 52]. Developing training programmes or guidelines/protocols for PROM collection processes for clinicians [10, 12, 37, 39, 40, 42, 43, 49, 53–57, 62, 64].	Lack of guidance or training for clinicians regarding the processes involved in PROM collection [10, 12, 37–39, 42, 49, 51, 53, 56, 64]. Lack of knowledge among clinicians in how to interpret PROMs [10, 38, 39, 42, 48, 49, 52, 55, 56, 64]. Some patient groups may have lower levels of digital literacy [40, 46, 49, 51, 52, 54, 55, 58, 60, 64, 65] or may have to use a proxy to complete the PROMs [40, 60, 65].
3. Social/professional role and identity	Clinicians feeling they have ownership over the system or personal responsibility for using PROMs, [37, 38, 55, 65] or if the application of PROMs was flexible with discretion given to the health professional [12, 56]. Engagement of managers and clinical leaders with the development and implementation of PROMs [12, 37, 47, 49, 54, 56, 64]. Engagement of patient representatives to include patient views of PROMs [41, 55, 64]. Presence of a clinician or manager as a ‘change champion’, or co-ordinator, who takes responsibility for PROMs implementation [12, 37, 39, 43, 44, 54, 64].	The burden of completing PROMs sometimes falls on a small number of staff or clinicians if the wider team are not participating fully, [12, 45, 48, 49, 54, 56, 64] leading to burn-out and stress [10, 37, 39, 43, 45, 56].
4. Beliefs about capabilities	Clinicians feeling comfortable using and interpreting PROMs [37]. Filling out the survey made patients feel more in control over the care they were receiving, and had an empowering effect [40, 42, 48, 56, 69].	Lack of confidence of clinicians in capabilities to contribute to the collection or interpretation of PROMs [41, 64]. Clinicians perceive that it may not be clinically possible to address the issues which were raised by patients through PROMs [4, 45, 49, 55]. Resistance to change and change fatigue among clinicians was found to be a barrier to implementation [41, 64].
5. Optimism	Clinicians convinced of high clinical value of PROMs supported implementation and were more open to accepting the use of PROMs in their role [37, 38, 42, 45, 49, 56, 68, 69]. Choosing PROMs which clinicians perceive as valid and reliable, [10, 12, 41, 54, 56] as well as choosing PROMs perceived as user friendly by clinicians The view among clinicians that PROMs will improve the culture between clinicians, managers and patients [64].	Perceived low clinical value of PROMs by clinicians, [10, 12, 37–39, 42, 45, 49, 51–54, 56, 68] including possible negative impacts on doctor patient relationship or quality of care, [10, 12, 37, 39, 41, 42, 47, 49, 53, 54, 56, 68] and concerns regarding credibility of data [55, 57]. Perceived low value of PROMs by patients—often seen as irrelevant to their care, leading to high attrition rates for follow ups [10, 12, 37–39, 42, 45, 48, 49, 52, 53, 56, 57, 68].
6. Beliefs about Consequences	Mechanisms which facilitate feedback of PROMs to patients and clinicians, [9, 37, 39, 40, 43] as well as having sufficient systems in place when issues are raised by PROMS [4, 5, 10, 37, 54, 56]. Beliefs among clinicians that PROMs use could save them time in their roles, [45, 49, 56] improve communication between staff and patients, [42, 45, 56] and encourage improved patient engagement [42, 45, 56].	Lack of feedback from clinicians to patients on PROM data [5, 64]. Beliefs among clinicians that using PROMs may have a detrimental impact for the patient-staff relationships, or quality of care [10, 12, 37, 39, 41, 42, 49, 53, 54, 56, 68]. Concerns among clinicians that using PROMs could result in management interference, [39] or data being misinterpreted by patients and the media leading to reputational damage[10]. Patients not aware of how PROMs are used or patients unhappy when PROMs are not considered enough in treatment plan[48, 54, 57, 64, 65].
7. Reinforcement	Sending one or multiple reminders helped patients to complete their surveys [9, 37, 39, 40, 43, 47, 64, 68]. Managers engaging with clinicians and other staff about PROM importance [12, 37, 45]. Regular meetings among clinicians to discuss PROMs data were facilitatory [38, 64]. Training programmes or clear guidelines/protocols for clinicians were reinforcing, [10, 12, 37–39, 41–43, 45, 48, 53–56, 58, 68] especially over multiple sessions[12, 38]	Financial incentives could encourage gaming of the system by clinicians, particularly when PROMs are imposed by an external agency[4, 10, 12, 56]. Poor, inconsistent or delayed communication or feedback of PROMs data to clinicians had a limiting effect, encouraging negative views about PROMs[4, 39, 49].
8. Intentions	Creation of organisational strategies or policies outlining the service’s intentions and actions related to PROMs [38, 42, 51]. Phased implementation of PROMs, involving an initial trial phase, with mechanisms to incorporate feedback from clinicians and patients [12, 45, 52].	Multiple initiatives involving PROMs with overlapping priorities can influence the sustainability of PROMs implementation[64].
9. Goals	Clearly setting out the organisational objectives of, and rationale in using PROMs, and communicating this to clinicians [39, 48, 56].	Implementing PROMs without clear aims or expections, [56, 64, 65] and financial goals [64]. Overlapping incentives and intentions leading to unclear expectations and uncertainty regarding the impact of PROMs initiatives [64].
10. Memory, attention and decision processes	Designing uniform systems that allow clinicians to access and use PROMs data in routine work [10, 12, 37, 41–43, 48, 49, 52, 54–56, 59, 66]. Aligning data collection with appointment schedules, [10, 37, 42, 56] or integrating PROM results into electronic health records can make it easier for clinicians to access and interpret PROMs data [10, 12, 39, 44, 45, 48, 49, 51, 52, 54, 55, 60, 62, 68]. Data presentation that ensures that interpretation was not time consuming for clinicians, with several reviews highlighting that graphical presentation of data was preferred by clinicians [12, 40, 48, 49, 53, 54, 56, 58, 60, 62, 65, 69]. Use of a single IT system, where you only need to log into a single database can improve adherence to PROM processes by clinicians [39, 48, 49, 52, 55]. Clinicians being able to access PROMs data at the individual patient level, [10, 12, 37, 38, 42, 44, 49, 52, 56, 69] and the data being in real time [40, 64]. Flexibility to adapt PROMs collection and feedback processes to be responsive to individual patient circumstances as some struggle to complete PROMs [10, 12, 37, 42, 46, 48, 49, 52, 56, 60, 62].	Collation of PROMs across multiple patients with the aim to monitor clinical performance, without mechanisms to review individual patient outcomes [10, 12, 42, 56]. Patients being too unwell, unwilling or lacking in capacity to complete PROMs [12, 37–40, 42, 48, 49, 51, 52, 55–57, 60, 62, 64]. Patients finding it too difficult to complete surveys independently, as they are too confusing, anxiety inducing, embarrassing, culturally insensitive or require advanced English language fluency [12, 38, 42, 44–50, 54, 55, 57, 58, 60, 62, 64, 65]. Poorly designed or drawn graphs were found to make it harder for clinicians to interpret PROM results, as was the inconsistent use of graph design [48, 55].
11. Environmental context and resources	Investment in health information technology systems to support PROMs collection such as electronic databases, web based platforms, and smart phone applications were enablers for clinicians and patients [9, 10, 12, 37–43, 46, 49, 52, 54–56]. A review of PROMs response rates found paper questionnaires had higher response rates than online questionnaires [9]. Other reviews emphasised flexible use of paper and electronic questionnaires increased response rates, [46, 47, 49, 50, 54, 57, 60, 64, 65]. ePROMs were highlighted to be more efficient at data collection, distribution and preserving data quality than paper based PROMs [46, 48–50]. Availability of sufficient administrative support for patients and clinicians, if there are issues in collecting PROMs, [10, 37–39, 47, 49, 54, 56, 60] and availability of sufficient statistical support for clinicians to appropriately analyse and interpret PROMs data [10, 37, 56]. Disease specific PROMs are sometimes found to be more clinically meaningful to clinicians, [48, 57, 64] and are and better received by patients [58].	The costs of implementing PROMs, such as license fees, with a lack of dedicated budget to support PROM implementation perceived as a barrier by clinicians and managers [37–40, 46, 49, 60, 68]. Clinicians feel they have limited capacity to respond to concerns raised by PROMs, particularly if there was no additional earmarked time created [37, 41, 42, 45, 54, 64]. Electronic systems that are difficult for patients or clinicians to use, [38, 47, 49, 54, 55]—technical issues included web browser incompatibility, password or software operational errors [9, 39, 41, 43, 48, 49, 51, 56]. Lack of time from both the patient and clinician perspective to engage in the collection and use of PROMS [10, 37, 39, 41–43, 47–49, 51, 54, 56, 60, 62, 64, 65]. Lack of integration of PROMs into electronic health records and other hospital systems created barriers to clinicians to engage with PROMs, [40, 48, 49] and lack of technical support for when problems arise [48, 55]. Lack of integration of PROMs into clinical work flow processes was also a barrier for clinicians [40, 48, 49, 55, 65].
12. Social influences	PROMs enacted in state or government policy can support implementation as clinicians and managers may be more actively motivated to engage with PROMs initiatives [39]. Aligning PROMs with clinical guidance so that clinicians perceived PROMs as part of their professional practice [42, 45]. Broad consensus and acknowledgement that PROMs are seen as valuable by clinicians and managers, [12, 38, 45] and sensitive leadership to motivate individuals and address concerns about the value of PROMs was a key enabler [37]. Maintenance of good relationships between the main facilitator, or champion, of PROM implementation and clinicians involved in PROMs processes, [37] especially with appreciation from management [56].	The perception by clinicians that an external agency was imposing PROMs on an organisation can act as a barrier to implementation [10]. Some clinicians thought that they did not get enough support from managers [3, 6].
13 Emotion	Clinicians need to feel they can influence the process of development and implementation of PROMs [37, 39]. Explaining the rationale and purpose of PROMs collection to patients, and reassurance regarding data protection and privacy processes in place to secure their data [12, 39–41, 43, 44, 49, 52, 56].	Clinicians fear that PROMs may have a detrimental impact on their relationship with patients, or quality of care [10, 12, 37, 39, 41, 42, 49, 53, 56, 68]. Clinicians fear that PROMs could be used for cost containment, or other unknown motives such as suspicion of micro-management, or judgement of work quality [39, 56]. Patient concerns around privacy and security of PROM data, [39–41, 43, 47, 49, 58, 64, 65] or the perception that PROM collection was impersonal or intrusive [10, 49, 56]. Patients fear being stigmatised due to mental health conditions leading to dishonesty in answering the questionnaires [58]. Clinicians worried that inadequate case mix control would bias the comparisons of healthcare providers and that the data doesn’t reflect practice [55].
14. Behavioural regulation	Developing an organisational plan by managers that included monitoring and evaluation of the processes involved in the use of PROMs, as well as regular feedback of PROMs data to clinicians supported implementation [37, 39, 40, 44, 46, 47, 54, 55, 59, 64, 65].	Using PROMs by managers as a performance management tool can contribute to poor engagement from clinicians in PROMs initiatives [4, 10]

### Knowledge

Knowledge is concerned with to what degree stakeholders such as patients, and clinicians are aware of PROMs. There was consensus among the reviews identified that low awareness among clinicians and patients about PROMs and their objectives was a significant barrier to implementation [10, 12, 37–39, 42, 49, 51, 56, 64]. This relationship was bi-directional, as improved awareness of PROMs was also frequently highlighted as an enabler for successful implementation of PROMs. To improve awareness, it was suggested that extensive consultation take place with all relevant stakeholders, including managers, clinicians, and patients, to improve awareness and develop a strategy for local implementation [12, 39, 41, 44, 52, 54].

### Skills

Skills reflects to what extent the abilities and competencies of clinicians to correctly interpret and respond to PROMs data influences implementation. Skill levels of clinicians was cited as a key barrier for implementation in many reviews [10, 38, 39, 42, 45, 48, 49, 51, 52, 55, 64]. Some reviews highlighted how a lack of guidance or training for clinicians regarding the processes involved in the use of PROMs collection was driving this [10, 12, 37–39, 42, 49, 51, 53, 56, 64]. Whereas, a common enabler identified among reviews was developing training programmes or guidelines for clinicians in terms of the processing involved in PROMs collection and interpretation [10, 12, 37, 39, 40, 42, 43, 49, 53–57, 62, 64]. A key strategy suggested within reviews to improve skills in relation to PROMs was to trial the use of a PROM to establish enablers and barriers to implementation that feed into the development of a wider PROM roll out [12, 39, 44, 52]. This can facilitate further training opportunities for clinicians, and allow staff to become more at ease with the PROM system [12, 45, 56, 68].

### Social/professional role and identity

Social/professional role and identity are related to how clinicians perceive their role or responsibilities regarding PROMs. Several reviews found that when clinicians felt they had ownership over the PROMs system or personal responsibility for using PROMs this was an enabler for implementation [37, 38, 49, 55, 65]. The importance of high level engagement of managers and clinical leaders with the development and implementation of PROMs was also emphasised [12, 37, 41, 49, 56, 64, 65]. The presence of a ‘change champion’, or co-ordinator, who takes responsibility for PROMs implementation was a significant enabler [12, 37, 39, 43, 44, 54, 64]. Although, other reviews were keen to emphasise that if the burden of completing PROMs falls on a small number of staff or clinicians and the wider team are not participating fully then this was a significant barrier [12, 45, 48, 49, 54, 56, 64]. In some cases where the burden of collection PROMs was falling upon one or two members of the team this can lead to burn-out and stress. [10, 37, 39, 43, 45, 56]

### Beliefs about capabilities

Belief about capabilities is related to how confident clinicians or patients feel regarding their capabilities to contribute to the collection or interpretation of PROMs. Antunes et al. and Glenwright et al. found that whether clinicians felt comfortable using PROMs was an enabler [37, 54]. This was a bi-directional relationship with lack of confidence in using PROMs cited as a key barrier in several reviews [38, 45, 49, 64]. Other reviews highlighted how clinicians feel they have limited capacity to respond to concerns raised by PROMs, particularly if there was no additional earmarked time created to do this [37, 41, 42, 45, 54, 64]. Other reviews highlighted the belief of clinicians that it may not be clinically possible to address the issues which were raised by patients through PROMs [45, 49, 55]. From the patient perspective, several reviews found patients engaging with PROMs had an increased feeling of control over the care they were receiving, and the process of filling out the surveys had an empowering effect [40, 42, 48, 56, 69].

### Optimism

Optimism is related to how optimistic clinicians and patients are regarding the value of PROMs. Perceived clinical value by clinicians was heavily cited by identified reviews as affecting their view on PROMs with perceived low clinical value persistently cited as a barrier to implementation of PROMs [10, 12, 37–39, 42, 45, 49, 51–54, 56, 68]. This perception was driven by many factors also highlighted below including concerns that PROMs may negatively impact the clinician- patient relationship or quality of care [10, 12, 37, 39, 41, 42, 49, 53, 56, 68]. This was shown to have a bi-directional relationship, with other reviews finding that clinicians convinced of the clinical value of PROMs supported implementation and they were more open to accepting the use of PROMs in their role [37, 38, 42, 45, 47, 49, 54, 56, 68, 69]. Choosing PROMs which clinicians perceive as clinically valid and reliable was identified as an enabler, [10, 12, 41, 54, 56] as well as choosing PROMs perceived as user friendly [10, 12, 54, 56]. The perceived low value of PROMs by patients, often being seen as irrelevant, [48, 57] or that they duplicate the clinical interview, [54] was another barrier to implementation.

### Beliefs about consequences

Belief about consequences is concerned with what clinicians believe happens in practice when using PROMs. Several reviews cited how clinicians had positive beliefs regarding the consequences of using PROMs, believing their use could save them time in their roles, [45, 49, 56] improve communication between staff and patients, [42, 45, 56] and encourage improved patient engagement [42, 45, 56]. As mentioned below, other reviews emphasised fears of clinicians that using PROMs may have a detrimental impacts to clinician-patient relationships, or quality of care [10, 12, 37, 39, 41, 42, 49, 53, 54, 56, 68]. Other reviews highlighted concerns from clinicians that using PROMs could result in management interference, [39] or data being misinterpreted by patients and the media leading to reputational damage [10]. Enablers that promoted positive beliefs about the consequence of using PROMs included mechanisms which facilitate feedback of PROMs to patients and clinicians [37, 39, 40, 43]. This was a bidirectional relationship as lack of feedback was also cited in one review as a barrier [49, 64]. Several reviews also found that having sufficient systems in place to identify and respond to issues raised by PROMs was an enabler [10, 37, 54, 56].

### Reinforcement

Reinforcement relates to the use of mechanisms such as incentives, penalties, reminders, or feedback to encourage the use of PROMs. Using financial incentives to encourage or reinforce the implementation of PROMs has produced mixed results in terms of supporting implementation of PROMs [10]. For example, Greenhalgh et al. found that the use of incentives could encourage gaming of the system by clinicians, particularly when PROMs are imposed by an external agency [10]. Communication and feedback were common features throughout. For example, sending one or multiple reminders to patients to complete their surveys, [37, 39–41, 43, 68] improved engagement and response rates. Persuading and engaging clinicians and other staff about the importance of PROMs acts as an enabler to encourage or reinforce the implementation of PROMs. Training programmes for clinicians provide an opportunity for this type of reinforcement as would other feedback opportunities such as regular clinical meetings to discuss PROMs data [10, 12, 37–39, 41–43, 45, 48, 53–56, 58, 68]. Conversely poor, inconsistent or delayed communication or feedback to clinicians had a limiting effect, encouraging negative views about PROMs, [39, 49] and sometimes clinicians were resistant to change and feedback regardless of attempts at reinforcement [10, 37, 39, 47, 56].

### Intentions

Intentions is concerned with the sustainability of plans or efforts to improve implementation of PROMs. Stability of intentions to support the implementation of PROMs can be enabled by the creation of clear organisational strategies or policies outlining the services’ intentions and actions related to PROMs [38, 42, 51]. This was sometimes termed good ‘cultural infrastructure’, where there was broad consensus and acknowledgement that PROMs are seen as valuable at the organisational level [38, 45, 64]. Conversely, multiple organisational initiatives with competing priorities and objectives were a barrier for sustainability of efforts targeted towards the implementation of PROMs [64]. Phased implementation of PROMs, involving an initial trial phase, with mechanisms to incorporate feedback from clinicians and patients supported sustainable efforts to implement PROMs [12, 45, 52].

### Goals

Goals is the use of any stated targets, aims or objectives to support the implementation of PROMs. The use of goals and targets was rarely discussed in reviews, although two reviews highlighted how clearly setting out the organisation objectives of, and rationale in using PROMs, and communicating this to clinicians, was a key enabler to support implementation [39, 48, 56]. Conversely, implementing PROMs initiatives without clear aims or objectives was perceived as a barrier to implementation due to challenges in establishing the impact of initiatives and unclear expectations for clinicians [56, 64, 65].

### Memory, attention, and decision processes

Memory, attention, and decision processes relates to how well systems that support the collection, processing, and interpretation of PROMs are designed to support the use of PROMs. Complex or difficult to use systems were persistently raised as a barrier for implementation by clinicians and patients throughout identified reviews [10, 12, 37, 40–42, 49, 53, 55, 56, 64, 65]. Designing systems that allow clinicians to access and use PROMs data in routine work were cited as enablers [10, 12, 37, 41–43, 48, 49, 52, 54–56, 59, 66]. For example, aligning data collection with appointment schedules, [10, 37, 42, 56] or integrating PROM results into electronic health records [10, 12, 39, 44, 45, 48, 49, 51, 52, 54, 55, 60, 62, 68]. Data presentation that ensures that interpretation by clinicians was not time consuming was a significant enabler, and several reviews highlighted that graphical presentation of data was preferred [12, 40, 48, 49, 53, 54, 56, 58, 60, 62, 65, 69]. The use of a single IT system, where clinicians only need to log into a single database could also improve adherence to PROM processes [39, 48, 49, 52, 55]. A further enabler was clinicians being able to access PROMs data at the individual patient level, especially if the data was in real time [10, 12, 37, 38, 42, 44, 49, 52, 56, 69]. In contrast, the collation of PROMs across multiple patients with the aim to monitor clinical performance, without mechanisms to review individual patient outcomes was perceived by clinicians as barrier [10, 12, 42, 56].

For patients, they were sometimes cited to be too unwell, unwilling or lacking in capacity to complete PROMs [12, 37–40, 42, 48, 49, 51, 52, 55–57, 60, 62, 64]. Some reasons include it being too difficult to complete surveys independently, or too confusing, anxiety inducing, culturally insensitive, or requiring too high a degree of comprehension or English language fluency [12, 38, 42, 44–50, 54, 55, 57, 58, 60, 62, 64, 65]. Potential solutions include improved guidance for patients, [39, 56] ensuring PROMs are designed in an inclusive and accessible manner, as well as ensuring that staff training includes how to best to help patients with the surveys [12, 53]. Moreover, some degree of adaptability of PROMs collection and feedback processes to suit individual patient circumstances has been cited by several reviews as an enabler [10, 12, 37, 42, 46, 48, 49, 56, 60, 62]. For example, some patients may struggle to complete PROMs, and require additional support, or seek a paper alternative if they have lower levels of digital literacy. [52]

### Environmental context and resources

Environmental context and resources relate to what infrastructure, human, or financial resources are available, and how this influences PROMs implementation. This domain was frequently commented upon across identified reviews, with many reviews citing how insufficient resources to implement PROMs into clinics was seen as a barrier by clinicians and managers [12, 40, 43, 45, 47, 49, 54–56, 60]. Investment in health information technology systems to support PROMs collection such as electronic databases, web based platforms, and smart phone applications was seen as a key enabler as they were perceived as reducing burden on clinicians and adminstrators [9, 10, 12, 37–43, 46, 49, 52, 54–56]. Although other reviews found that electronic systems are difficult for patients or clinicians to use, [38, 47, 49, 54, 55] and technical issues cited included web browser incompatibility, challenges remembering passwords, and software operational errors [39, 41, 43, 48, 49, 51, 56]. A review of published response rates of PROMs with at least two follow-up time points found that paper questionnaires had higher response rates than online questionnaires [9]. Other reviews emphasised that a mix of paper and electronic questionnaires may be necessary to support implementation in a way which does not discriminate against populations with lower levels of digital literacy [44, 46, 49, 50, 52, 54, 57, 60, 64, 65]. In terms of human resources, several reviews cited the availability of sufficient administrative support for patients and clinicians, if there are issues in collecting PROMs, [10, 37–39, 47, 49, 54, 56, 60] and the availability of sufficient statistical support for clinicians to appropriately analyse and interpret PROMs data as enablers [10, 37, 56]. Lack of time from both the patient and clinician perspective to engage in the collection and use of PROMs has been cited as a barrier [10, 37, 39, 41–43, 47–49, 51, 54, 56, 60, 62, 64, 65].

### Social influences

Social influences relate to how relationships, social pressure, or group norms impacts implementation of PROMs. An important social influence was that of governance and policy. Gelkopf et al. found that PROMs work best when enacted in state or government policy as clinicians and managers may be more actively motivated to engage with PROMs initiatives [39]. Conversely, Greenhalgh et al. found that the perception among clinicians that an external agency was imposing PROMs can act as a barrier to implementation [10]. Aligning PROMs with clinical guidance was seen as an enabler as clinicians perceived PROMs as part of their professional practice [42, 45]. Antunes et al. found that having sensitive leadership to motivate individuals and address concerns about the value of PROMs was a key enabler [37]. Similarly, the maintenance of good relationships between the main facilitator, or champion, of PROM implementation and clinicians and other staff was important, [37] especially when good feedback and appreciation was provided by management [56].

### Emotion

Emotion related to any concerns, fears, or anxieties that clinicians or patients may have regarding the use of PROMs. Several reviews found a key barrier to implementing PROMs was clinicians concerned that PROMs may have a detrimental impact on their relationship with patients, or quality of care [10, 12, 37, 41, 42, 49, 53, 56, 68]. Boyce et al., and Gelkopf et al. also found fears that PROMs could be used for cost containment, or other unknown motives, was another concern raised by clinicians [39, 56]. To address these concerns, clinicians needed to feel they were involved and can influence the process of development and implementation of PROMs [37, 39]. From the patient perspective, several reviews found that concerns around privacy and security of PROM data, [39–41, 43, 47, 49, 58, 64, 65] or the perception that PROM collection was impersonal or intrusive, [10, 49, 56] were barriers to implementation. Addressing these concerns requires explaining the rationale and purpose of PROMs collection, and reassurance regarding data protection and privacy processes in place to secure their data [12, 39–41, 43, 44, 49, 52, 56].

### Behavioural regulation

Behavioural regulation is the use of mechanisms that monitor, or measure actions related to PROMs implementation. Many reviews found that developing an organisational plan that included monitoring and evaluation of the processes involved in the use of PROMs, as well as regular feedback of PROMs data to clinicians supported implementation [37, 39, 40, 44, 46, 47, 54, 55, 59, 64, 65]. However, some reviews found that absent or delayed feedback of PROMs data to clinicians or clinicians not looking at PROMs data was a barrier to implementation [39, 49, 64]. Another review concluded that using PROMs as a performance management tool can also prove to be a barrier to securing the engagement of clinicians, particularly if the PROMs data was not seen as credible or reflective of the skills or capabilities of individual clinicians [4, 10].

## Discussion

### Summary of findings

Drawing upon the TDF, this umbrella review has identified key enablers and barriers applicable to PROMs implementation in routine clinical practice. We focused on categorising enablers and barriers to PROMs implementation according to different domains which influence the behaviours, perspectives, and beliefs of the different stakeholders involved in the use of PROMs in routine clinical practice. Knowledge, skills, social/professional role and identify, belief about consequences, memory, attention and decision processes, environmental context and resources, and social influences, were all prominent domains in our thematic analysis. From the patient perspective, barriers to PROMs implementation included low awareness of PROMs, perceived low value of PROMs, PROMs with surveys that were too difficult to complete independently, anxiety inducing, or culturally insensitive, concerns around privacy and security of PROM data, and the perception that PROM collection was impersonal or intrusive. Enablers to strengthen PROMs implementation included consultation with patients prior to implementation, staff training regarding strategies to help patients fill in surveys, adaptability of PROMs format, collection, and feedback processes to suit individual patient needs, sending reminders to complete PROMs, and explaining the rationale and purpose of PROMs collection, and reassurance regarding data protection and privacy processes.

From the clinician perspective, barriers to PROMs implementation included low awareness of PROMs, poor skills and capabilities related to using and interpreting PROMs, lack of ownership or personal responsibility related to PROMs, perceived low clinical value of PROMs, complex or difficult to use PROMs systems, absent or delayed feedback of PROMs data, limited capacity or capabilities to respond to issues raised by PROMs, and concerns PROMs may have detrimental impacts on patient relationships or be used as a performance management tool. Common enablers to PROMs implementation included training programmes or guidelines related to PROMs collection and interpretation, choosing PROMs that clinicians perceive as clinically valid, mechanisms to facilitate feedback of PROMs data, sufficient systems to respond to issues raised by PROMs, designing systems to access PROMs data in routine work, graphical presentation of PROMs data, accessing PROMs data at the individual patient-level, availability of sufficient administrative and technical support, and involvement of clinicians in PROMs development and implementation. From a system-level perspective, there were many enablers and barriers to PROMs implementation that influenced several stakeholders involved in PROMs implementation. Barriers to PROMs implementation included insufficient resources to implement PROMs into clinics, and multiple organisational initiatives with competing priorities and objectives. Enablers included high-level engagement from leadership and managers, developing an organisational plan with clear goals and targets, designating ‘change champions’ or co-ordinators with responsibility for PROMs implementation, and broad consultation with relevant stakeholders involved in PROMs implementation to address concerns and outline expectations. There were mixed implications of using financial incentives or enacting PROMS within state or government policy to support PROMs implementation.

### Strengths and limitations

The major strength of this umbrella review is that it summarises a large body of literature on the enablers and barriers to supporting the implementation of PROMs thematically using an implementation science framework that captures the perspective of multiple stakeholders involved in PROMs implementation in routine clinical practice. While comprehensive, this umbrella review does come with limitations. First, our search scope was confined to three electronic databases—MEDLINE, EMBASE, and PsychInfo, and our search scope could have been strengthened through other databases such as the Healthcare Administration Database, [70] or Web of Science [71]. Second, we acknowledge it is possible that other research teams may have reached different conclusions regarding the clustering of enablers and barriers as it was primarily based on iterative discussions between only two reviewers. Although, we were limited by resource constraints, and we were unable to involve additional authors within our data extraction process. Third, our eligibility criteria, emphasizing English language reviews, might have led to oversight of insights from non-English articles. Fourth, although we leverage the TDF to thematically summarise findings, our approach lacks a systematic or quantitative analysis of these domains contained within the framework. Hence, it is essential to view our review solely as offering a snapshot of available evidence concerning enablers and barriers to PROMs implementation. Fifth, we do not categorise our findings or exclude any review based upon quality assessment of identified reviews. While this was a deliberate decision to increase the comprehensiveness of our findings, we cannot indicate which findings were from reviews with high or low quality. Sixth, we do not categorise our findings by clinical context or speciality which may have been useful to stakeholders working in specialised settings. However, this was not possible due to the broad range of different clinical settings identified in reviews. Lastly, the TDF has been criticised as not comprehensively capturing barriers to behaviour change related to clinician knowledge and perception when compared to the CFIR [14]. It has also been emphasised that the TDF is more suited to categorising data from interviews and focus groups, rather than from other collection methods such as surveys or observation [35]. However, a review of different frameworks used to evaluate approaches to PROMs implementation acknowledged that the TDF and CFIR frameworks produced similar results, and no single framework comprehensively captured all nuances related to implementation. [15]

### Comparison with previous literature

This umbrella review has identified many enablers and barriers to PROMs implementation in routine clinical practice that have been highlighted in previous key reviews focused on this issue. Greenhalgh et al. focused on how to feedback aggregate and individual-level PROMs data to improve patient care, and emphasised the use of credible data, data which identifies clear problems, and timely feedback [10]. These issues were also discussed in many other reviews we identified [10, 12, 37, 39, 41, 42, 47, 49, 53, 54, 56, 68]. Similar to Greenhalgh et al. [10], we also identified findings from reviews that emphasised the importance of designing systems to access PROMs data in routine work, [10, 12, 37, 41–43, 48, 49, 52, 54–56, 59, 66] graphical presentation of PROMs data, [12, 40, 48, 49, 53, 54, 56, 58, 60, 62, 65, 69] and clinician ownership and involvement in the development and implementation of PROMs initiatives [37, 38, 55, 65]. We identified other enablers to improving use of PROMs data to improve patient care including organisational strategies with clear objectives, and expectations for relevant stakeholders, [39, 48, 56, 64, 65] and the presence of of a clinician or manager as a ‘change champion’, or co-ordinator, who takes responsibility for PROMs implementation [12, 37, 39, 43, 44, 54, 64].

Gibbons et al. found evidence that the use of PROMs in routine clinical practice improves quality of life, and increases patient-clinician communication, diagnosis of disease, and disease control [11]. Despite this, we identified several reviews that emphasised that patients, and clinicians, remain unconvinced regarding the value of using PROMs in routine clinical practice [10, 12, 37–39, 42, 45, 48, 49, 51–54, 56, 57, 68]. Other reviews emphasised that clinicians may have concerns that PROMs use could negatively impact the patient-clinician relationship [10, 12, 37, 39, 41, 42, 47, 49, 53, 54, 56, 68]. Therefore, it is important when designing PROMs initiatives that broad consultation with relevant stakeholders takes place prior to implementation to address such concerns, and inform them of positive evidence regarding the use of PROMs in routine clinical practice.

Foster et al. highlighted several barriers to PROMs implementation in routine clinical practice that were also highlighted in reviews we identified including resource constraints, [37–40, 46, 49, 60, 68] questionnaire complexity, [12, 38, 42, 44–50, 54, 55, 57, 58, 60, 62, 64, 65] challenges in interpreting data, [10, 38, 39, 42, 48, 49, 52, 55, 56, 64] lack of time to engage with PROMs processes, [10, 37, 39, 41–43, 47–49, 51, 54, 56, 60, 62, 64, 65] and professional reluctance and concerns regarding the credibility and value of PROMs data [10, 12, 37–39, 42, 45, 49, 51–54, 56, 68]. Enablers also identified within our umbrella review and Foster et al. include broad stakeholder engagement throughout implementation, [12, 39, 41, 44, 52, 54] adaptability of PROMs processes, [10, 12, 37, 42, 46, 48, 49, 52, 56, 60, 62] training for clinicians, [10, 12, 37, 39, 40, 42, 43, 49, 53–57, 62, 64] and designating implementation leads to oversee implementation. [12, 37, 39, 43, 44, 54, 64] We identified several other enablers including sending one or multiple reminders to patients to complete their surveys, [9, 37, 39, 40, 43, 47, 64, 68] designing uniform systems that allow clinicians to access and use PROMs data in routine work, [10, 12, 37, 41–43, 48, 49, 52, 54–56, 59, 66] and regular feedback of PROMs data to clinicians [37, 39, 40, 44, 46, 47, 54, 55, 59, 64, 65]. We also identified other important barriers including patient concerns around privacy and security of PROM data, [39–41, 43, 47, 49, 58, 64, 65] the perception that PROM collection was impersonal, [10, 49, 56] or patients being too unwell, unwilling or lacking in capacity to complete PROMs. [12, 37–40, 42, 48, 49, 51, 52, 55–57, 60, 62, 64]

### Policy implications and directions for future research

From a policy viewpoint, encouraging the implementation of PROMs in routine clinical practice needs to be integrated into other health policy initiatives. For example, policies that aim to promote digitalisation of health can ensure the integration of PROMs systems into electronic health record platforms so they are easily accessible to patients and clinicians [72, 73]. Moreover, workforce strategies need to mobilise resources for PROMs training programmes, facilitate opportunities for clinicians or managers to become implementation leads or “change champions” within healthcare organisations, and earmark time for clinicians to respond to issues raised by PROMs [74]. To enhance the likelihood of successful implementation of PROMs, collection methods for PROMs need to be co-designed with patients to improve their usability and uptake [75, 76]. Educational materials need to be produced for patients that explain the value of PROMs, how to engage with PROMs, and measures in place regarding data protection and privacy. Crucially, PROMs need to be framed as a tool to promote patient centeredness and empowerment as well as an effective mechanism to enhance patient-clinician communication [77]. PROMs processes also need to be flexible and adapted to individual patient needs, for example providing paper format PROMs for certain patients who may experience digital exclusion, or availability of PROMs in different languages [78]. More research is needed to understand the applicability of disease-specific and non-specific PROMs in different clinical and cultural contexts, and implications for broader policy and decision making. While some clinicians emphasise the need for disease-specific PROMs for their speciality areas, this has trade-offs including increasing complexity, and challenges in evidence synthesis and meta-analysis [79]. There have been movement towards developing international standards for PROMs, specifically the National Institutes of Health Patient-Reported Outcomes Measurement Information System (PROMIS) [80]. Assuming clinicians and patients become familiar with these standards over time, this would help overcome some of the implementation barriers described in this review including lack of awareness, understanding, and interpretability of PROMs scores [81]. However, to ensure such systems are used widely in routine clinical practice there needs to be buy-in from speciality groups, patient organisations, and national healthcare bodies [82]. An initial step to help build consensus on the use of PROMs systems such as PROMIS in routine clinical practice would be to fund feasibility and acceptability studies in different clinical contexts [83]. We did not identify detailed information on how the use of incentives or penalties influence PROMs implementation in our reviews. Two reviews discussed how mandating PROMs as part of state policy can influence implementation, with Gelkopf et al. concluding this can support implementation and Greenhalgh et al. concluding this can create a perception that PROMs are externally imposed and therefore reduce engagement from clinicians [10, 39]. Greenhalgh et al. also discussed how there remains considerable variation in PROMs participation rates across providers in England despite the existence of financial incentives to engage with PROMs processes [10]. Therefore, the impact of different types of incentives and penalties on PROMs implementation in routine clinical practice remains an area for future research which needs to be addressed.

## Conclusion

The integration of PROMs in clinical practice holds the promise of profoundly improving healthcare quality and empowering patients in their healthcare pathways. Despite this, many PROMs initiatives that aim to integrate PROMs within routine clinical practice fail to achieve their objectives or suffer from poor engagement from clinicians and patients. Our review has exposed the complexities of PROMs implementation and potential pitfalls and solutions to challenges experienced from the patient, clinician, and system-level perspectives. In doing so, this review offers guidance to policy-makers seeking to seamlessly and sustainably integrate PROMs into routine clinical practice.

## Author contributors

MA, AS, and JF: designed the study. MA and AS: conducted title and abstract screening and data extraction until December 2020, this was later updated by MA and EW to June 2023. MA and EW performed the quality assessment. MA, RVK, and AS: undertook the initial drafting of the manuscript. All authors commented and edited iterative drafts of the manuscript.

## Supplementary Information

Below is the link to the electronic supplementary material.

Supplementary file1 (DOCX 56 KB)

Supplementary file2 (XLSX 23 KB)

## Data Availability

Not applicable.
