# Supplementary material for: Understanding factors impacting patient-reported outcome measures integration in routine clinical practice: an umbrella review
Source: Qual Life Res. 2024 Jul 18;33(10):2611–29. doi: 10.1007/s11136-024-03728-7 (PMC11452453; doi:10.1007/s11136-024-03728-7)
Supplement: Supplementary file 1 — Supplementary file1 (DOCX 56 KB) [file 11136_2024_3728_MOESM1_ESM.docx]

*Supplementary Material Table 1: Reviews identified focused on PROM implementation*

| Review | Focus | Type of review | Approach to data synthesis | Perspectives captured | Quality  Assessment Issues | Articles Identified |
| --- | --- | --- | --- | --- | --- | --- |
| Anderson et al 2020^52^ | PROMs and parent proxy reported outcomes in paediatric settings | Systematic review | Narrative synthesis | Patients, and clinicians | Unclear research question  Excluded grey literature  No quality assessment | 32 |
| Antunes et al 2014^37^ | Implementing PROMs in palliative care clinical practice | Systematic review | Narrative synthesis | Patients, clinicians, and managers | None | 31 |
| Bantug et al 2016^53^ | Use of graphical displays of PROMs in clinical practice | Integrative review | Thematic synthesis | Patients, and clinicians | Unclear research question  Only used key word search in Ovid  No quality assessment | 9 |
| Blood et al 2021^46^ | Implementing patient reported outcome measures in melanoma clinical quality registries | Systematic Review | Narrative synthesis | Clinicians | None | 14 |
| Boyce et al 2014^56^ | Experience of professionals using PROMs to improve healthcare quality | Systematic review | Thematic synthesis | Clinicians | None | 16 |
| Briggs et al 2020^38^ | Implementing PROMs in outpatient rehabilitation settings | Systematic review | Framework synthesis using Consolidated Framework for Implementation Research (CFIR) | Patients, clinicians, and managers | None | 10 |
| Campbell et al 2022^57^ | The benefits and limitations of using patient-reported outcome measures in clinical practice | Systematic Review | Thematic synthesis | Patients, and clinicians | None | 50 |
| Carfora et al 2022^58^ | Experience of patients of patient-reported outcome measures in clinical care | Systematic review and qualitative meta-synthesis | Meta-ethnography | Patients | None | 14 |
| Dorr et al 2022^55^ | Improving the quality of healthcare trajectories using patient reported outcomes | Systematic review | Quantitative data: Narrative synthesis  Qualitative data: Thematic synthesis | Patients, clinicians, and managers | None | 16 |
| Easpaig 2020 et al^45^ | Attitudes of health professionals to PROMs in oncology | Systematic review | Sentiment analysis followed by thematic synthesis | Clinicians | None | 34 |
| Eijsink et al 2023^59^ | The implementation of patient reported outcomes in western countries for hospital based interventions | Scoping review | Narrative synthesis | Patients, and clinicians | Unclear research question  Excluded systematic reviews  No quality assessment | 20 |
| Foster et al 2018^12^ | Implementing PROMs in healthcare organisations | Umbrella review | Framework synthesis using Consolidated Framework for Implementation Research (CFIR) | Patients, clinicians, and managers | None | 6 |
| Garcia Abejas et al 2023^47^ | The use of patient reported outcome measures to improve the quality of life of patients with lung cancer | Scoping review | Narrative synthesis | Patients, clinicians, and managers | Excluded grey literature  No quality assessment | 12 |
| Gelkopf et al 2022^39^ | Goals, implementation, setting, measurement characteristics and barriers of using PROMs in mental health services | Systematic Review | Narrative synthesis | Patients, clinicians, and managers | Excluded grey literature  No quality assessment | 103 |
| Glenwright et al 2023^54^ | The facilitators and barriers to implementing electronic patient reported outcome measures in healthcare | Systematic review | Framework synthesis using Consolidated Framework for Implementation Research (CFIR) | Patients, clinicians, and managers | Excluded studies with <75% usage of ePROMs | 24 |
| Greenhalgh et al 2017^10^ | Collation, interpretation and use of PROMs to improve patient care | Realist review | Realist synthesis | Patients, clinicians, and managers | None | 36 |
| Greenhalgh et al 2018^4^ | The use of aggregated patient-reported outcome measures data to stimulate health care improvement | Realist  review | Realist synthesis | Patients, clinicians, and managers | None | 68 |
| Greenhalgh et al 2018^5^ | The use of patient reported outcome measures to support clinician-patient communication and patient care | Realist  review | Realist synthesis | Patients, and clinicians | None | 39 |
| Howell et al 2015^42^ | Use, impact on health outcomes, and implementation of PROMs in cancer care | Scoping review | Narrative synthesis | Patients, and clinicians | Unclear research question  No quality assessment | 30 |
| Hyland et al 2022^60^ | Implementing patient reported outcomes for underrepresented patients in routine clinical practice | Scoping Review | Framework synthesis using Proctor et al 2011 taxonomy^61^ | Patients, and clinicians | Excluded grey literature | 28 |
| Kroenke et al 2022^62^ | Choice and use of patient reported outcome measures in clinical practice | Systematic Review | Framework synthesis using Terwee et al 2018^63^ | Patients, clinicians, and managers | Unclear research question  Excluded grey literature  No review of reference lists  No quality assessment | 27 |
| Kynoch et al 2022^64^ | The use of patient reported data in acute healthcare | Scoping Review | Narrative synthesis | Patients, clinicians, and managers | No quality assessment | 86 |
| Laitio et al 2023^48^ | The use of patient reported outcome meausres and the perceived barriers and facilitators | Systematic mapping study | Thematic synthesis | Patients, clinicians, and managers | No quality assessment | 17 |
| Lutz et al 2022^40^ | The use of patient reported outcome measures in oncology | Systematic review | Narrative synthesis | Patients, and clinicians | None | 25 |
| Minvielle et al 2023^65^ | Patient reported outcome measures within oncology for health policy purposes | Scoping review | Thematic synthesis | Patients, clinicians, and managers | No quality assessment | 61 |
| Nguyen et al 2021^49^ | The barriers to using patient reported outcome measures in routine cancer care | Systematic Review | Narrative synthesis | Patients, clinicians, and managers | No quality assessment | 14 |
| Saleem et al 2023^50^ | Digitalising patient reported outcome measures and collection in orthopaedic trauma | Scoping review | Narrative synthesis | Patients | No quality assessment | 6 |
| Scheibe et al 2020^44^ | Implementing PROMs in in routine cancer care | Systematic review | Narrative synthesis | Patients, clinicians, and managers | Unclear research question  No quality assessment | 29 |
| Silveira et al 2022^41^ | The use of patient reported outcomes in oncology | Systematic review | Narrative synthesis | Patients, clinicians, and managers | None | 83 |
| Sokas et al 2022^51^ | Implementing patient reported outcome measures in surgical practice | Scoping Review | Framework synthesis using Proctor et al 2011 taxonomy^61^ | Clinicians, and patients | No quality assessment | 16 |
| Sørensen et al 2019^66^ | The use of PROMs in clinical decision making for hip and knee osteoarthritis | Scoping review | Narrative synthesis | Clinicians | Unclear research question  Excluded systematic reviews and grey literature  No quality assessment | 349 |
| Van Egdom et al 2019^43^ | Implementing PROMs in clinical breast cancer care | Systematic review | Narrative synthesis | Patients, clinicians, and managers | None | 34 |
| Wang et al 2020^9^ | An overview of PROM response rates in registry-based studies, and approaches to data collection | Systematic review | Narrative synthesis | Patients | No quality assessment | 28 |
| Yang et al 2018^67^ | The impact of PROMs on patient-clinician communication in oncology | Realist review | Realist synthesis | Clinicians, and patients | None | 43 |
